# ChatGPT applications in academic writing: a review of potential, limitations, and ethical challenges

**DOI:** 10.5935/0004-2749.2024-0269

**Published:** 2024-12-26

**Authors:** Yuanyuan Liu, Wenting Kong, Kaygac Merve

**Affiliations:** 1 School of Foreign Studies, China University of Petroleum (East China), Qingdao, Shandong, China; 2 School of International Education, China University of Petroleum (East China), Qingdao, Shandong, China

**Keywords:** ChatGPT, Writing, Authorship, Ethics, Data accuracy, Plagiarism, Students, Bias

## Abstract

This article systematically reviewed 327 documents in the core collection of the Web of Science database regarding ChatGPT applications in the writing domain. This study aimed to comprehensively assess the latest progress and potential applications. ChatGPT demonstrates significant potential in overcoming writing anxiety, improving writing efficiency, generating initial scientific papers, and assisting researchers and students in giving feedback. However, it still faces significant challenges in data accuracy and the ethics of generated content, including inaccurate or outdated information, plagiarism risks, gender or race biases, etc. Authorship verification is particularly important for academic writing and publishing as it relates to objectivity, accuracy, and fairness. Future studies need to explore how to address these challenges through improvements at the technical and policy levels, ensuring that ChatGPT promotes the sustainable development and application of academic writing while adhering to ethical standards.

## INTRODUCTION

Since the inception of ChatGPT, there has been increased interests in the potential impact across various sectors^(1^-^3)^. This surge has catalyzed extensive research by scholars from domestic and international spheres, transcending far beyond the boundaries of computer science^(4)^ to encompass disciplines such as education and psychology, with a particular focus on the realm of academic writing.

ChatGPT, with its impressive contextual understanding and coherent dialogue capabilities, is a revolutionary writing aid for authoring academic articles^(5)^. It can become a valuable tool and transform the writing process^(6)^. In a global postdoctoral survey by *Nature*, approximately one-third of respondents utilized artificial intelligence (AI) for refining texts, writing code, or organizing literature^(7)^.

Despite its immense potential, it still faces challenges, such as authorship issues, data inaccuracies, and biases^(4)^. Addressing ethical and legal concerns while maintaining academic integrity and data reliability is crucial. This article analyzed ChatGPT applications for the past 2 years, focusing on research hotspots and concerns. By examining research trends, this article unveiled the practical applications of ChatGPT in the writing domain and provided constructive suggestions for future development.

## METHODS

### Research aims

This scoping review aimed to appraise the empirical literature on the application and provide directions for future investigation. This review sought to explore (a) the landscape of studies involving ChatGPT applications, (b) the impact and consequences of studies involving AI-facilitated writing, and (c) significant challenges in terms of data accuracy and ethics.

### Design

A systematic scoping review involves the following steps: (a) identification of the area of interest; (b) systematic literature search; (c) study selection, and (d) collation and reporting the results.

This article utilized core collections from databases such as Web of Science, ScienceDirect, and SpringerLink using “ChatGPT”, “chatgpt”, and “chat gpt” as the core search terms for topic retrieval. This study selected journal articles, conference papers, and book citation index sublibraries from multiple sources, including SSCI, A&HCI, SCI, ESCI, CPCI, and BKCI, to ensure comprehensiveness and integrity. The search period was set from the release of ChatGPT until May 2024.

Inclusion criteria: (a) involves ChatGPT applications in the writing domain; (b) was published in a credible academic source; (c) was primary research; and (d) publication date is within the time range. Exclusion criteria: (a) irrelevant or commentary literature; (b) out of the publication date range; and (c) duplicated or lack of focus study. Utilizing EndNote automation tools to assist in screening, 327 effective pieces were identified as analysis samples. This study focused on refining research hotspots and did not involve quantitative trend analysis, therefore, bibliometric analysis methods were not used.

## RESEARCH HOT TOPICS

The academic community has shown a strong research interest in ChatGPT applications across multiple writing domains. These areas include, but are not limited to, intelligent writing, academic paper assistance, overcoming writing anxiety, providing immediate feedback, and quality assessment. Existing studies also focused on certain ethical and technical challenges, such as issues of authorship, potential errors, and inherent biases within algorithms.

### 1. Implementation of intelligent writing

ChatGPT has become increasingly important in scientific writing^(8^,^9)^. AI-based literature search, analysis, and synthesis tools can assist authors in effective and efficient writing. Compared to humans, AI has demonstrated higher creative potential in divergent thinking tests^(10)^.

#### 1.1. Overcoming writing anxiety

AI-driven writing assistance tools offer an interactive and customized approach to enhance writing skills and motivation^(11^,^12)^, help writers overcome psychological barriers such as lack of motivation and writing anxiety^(13)^, and explore new avenues for writing practices, proving their value as precious assistance tools^(14^,^15)^.

Chatbots are equipped with complex algorithms and functionalities^(16^-^18)^. ChatGPT expands students’ linguistic choices, provides immediate feedback, and helps alleviate writing anxiety^(12)^ and psychological stress during the learning process^(19)^. It can recommend vocabulary and phrases and perform text rephrasing, rewriting, and proofreading ^(20)^ to enhance the overall quality and structure of writing^(6)^. By offering a broad language repertoire^(19^,^21)^, ChatGPT encourages students to think from multiple perspectives. ChatGPT incorporates an automatic writing evaluation system, providing meaningful guidance and substantive feedback before, during, and after writing^(15)^, enhancing learner motivation^(13^,^22)^ and increasing the enjoyment of writing. Students using AI writing tools had higher engagement scores than the control group^(23)^, and performance was superior^(24)^, suggesting promising potential for stimulating motivation and capability.

For nonnative English speakers, ChatGPT is particularly beneficial^(25)^, enabling them to focus on higher-level thinking and essentially eliminating barriers to English writing.

Previous research also revealed problems, such as the lack of human review, overreliance, and limitations in language diversity. Future research could consider combining the strengths of AI and human review using ChatGPT as an assistive tool with teachers or professionals conducting final reviews and edits. Meanwhile, teachers should guide students to cultivate independent thinking and creativity. Developing more diverse language models can meet the needs of different languages and cultures.

#### 1.2. Content generation, feedback, and revision

ChatGPT aids academic writing at multiple levels by serving as an effective writing assistant, encompassing literature reviews, summaries, and detailed descriptions^(6^,^26^-^28)^; enhancing the efficiency by providing drafts, checking language, grammar, and spelling^(29^,^30)^; offering guidance on academic writing form^(9)^ and style^(26)^; identifying potential errors, inconsistencies, or gaps in arguments^(26)^; providing instant feedback and suggestions for revision^(31)^; enhancing manuscript quality^(32)^; assisting in refining text^(7)^; improving readability, clarity, and accuracy^(33)^; and aiding in tasks related to formatting, language, and content review^(6)^. ChatGPT effectively explains well-known concepts, translates between languages, adjusts text style and tone, and perfects writing mechanics to enhance efficiency and quality.

ChatGPT has been employed in journalism for information analysis, content extraction, audience research, automatic copywriting^(34)^, and the generation of news reports, significantly reducing the creative cycle. Combined with human critique, it can elevate writing to a new level^(6)^. Journalists have recognized the significant risks, including inaccuracies and the lack of empathy.

Previous research showed shortcomings, particularly in in-depth analysis, personalized services, creativity enhancement, ethics, and privacy protection. Future research must evaluate AI feedback effects multidimensionally, develop personalized and customized services, enhance creativity and stylistic diversity under AI assis-tance, strengthen ethics and privacy protection, and gain a deeper understanding of human writing behavior through interdisciplinary collaboration.

#### 1.3 Assisting in scientific paper writing

ChatGPT assists in vocabulary selection and structure organizing, the generated text is formal and objective, and readability is enhanced^(35)^. ChatGPT aids researchers in shortening the time for data analysis and publication of scientific knowledge^(36)^, and identifying research questions and significance^(25)^. ChatGPT contributes to better expressing human thoughts and perspectives^(37)^, allowing intellectual efforts to focus more on critical thinking and analytical writing^(20^,^38)^ rather than merely expending energy on forming preliminary ideas.

Researchers also frequently utilize ChatGPT to predict development trends in specific scientific fields^(1)^, achieve efficient literature search and information extraction, identify research gaps and data training, and monitor the latest publications^(39)^. ChatGPT assists in analyzing qualitative data^(40)^, rapidly processing large qualitative datasets, and saving researchers’ time.

ChatGPT creates high-quality conference summaries using virtual datasets without apparent errors^(41)^. It simplifies lengthy documents, reduces repetition, polishes or assists scientists with limited English skills by offering language advice, and increases participation in global academic dialogue^(42^-^44)^. It can even enable nonscientists to write satisfactory papers and handle reviewers’ comments^(45)^, with some editorials or letters written using ChatGPT already published^(46)^. Comparison with baseline models indicates text authenticity and verifiability, reducing factual errors^(47)^.

Most studies focused on improving writing styles, although the potential role of AI in promoting scientific innovation and theoretical construction, ethics, and reliability has been insufficiently explored. How researchers interact with ChatGPT and how this interaction affects the writing process and outcomes are lacking.

Subsequent research should concentrate on establishing ethical guidelines and legal frameworks for using AI to assist scientific writing. Furthermore, designing long-term tracking studies to evaluate the impact of ChatGPT on the research ecosystem is essential. Continuous optimization of ChatGPT algorithms to reduce the generation of incorrect information, enhance its accuracy and reliability within specialized fields, and protect sensitive data of individuals and institutions are the key directions for future research.

#### 1.4. Peer review capabilities

ChatGPT assists in initial screening, identifying potential issues related to ethics, integrity, or quality^(37)^, providing rapid feedback, detecting possible defects, alleviating the burden on human reviewers, and acce-lerating publication time^(48^,^49)^. ChatGPT consistently follows preset standards and guidelines, reducing the subjective biases and variability that human reviewers might introduce. Research showed that the perspectives raised by ChatGPT align with those of human reviewers, with overlap existing^(50)^, and it can handle reviewer comments in 55% of the cases^(45)^. Controlled experiments also indicated that writing feedback generated by ChatGPT is indistinguishable from experts.

ChatGPT lacks the professional knowledge to assess the scientific validity or accuracy of complex research outcomes, has difficulties vetting highly specialized scientific topics^(51)^, exhibits omissions and biases^(50)^, and struggles to produce precise information in fields where little research exists^(52)^. The level of review articles written by ChatGPT is insufficient to meet expert needs. Half of the population expresses ethical concerns about plagiarism^(53)^ in scientific research. It further narrows the scholarly knowledge space, hinders the expression of diverse ideas, and impairs the integrity of the academic environment monitoring function^(54)^.

The automation of the peer review process is far from reality. It cannot simply replace the tangible and emotional quality of human experience. Some scholars insisted on strictly prohibiting its usage in the peer review ^(55)^. Research showed that peer reviewers can only identify 63% of the abstracts written by ChatGPT, misjudging them as genuine^(8)^. Relying solely on naturalness, fluency, and writing patterns, reviewers cannot distinguish the differences, and some content may include intellectual property without explicit permission or proper citation.

Scholars have not fully assessed ChatGPT applications in peer review on the academic publishing ecosystem, including impacts on academic quality, innovation, and knowledge dissemination. How to ensure transparency, fairness, and auditability of AI applications and how to better combine human reviewers’ intuition, experience, and ethical judgment while ensuring the efficacy of AI technology are crucial. Comparative studies on the adaptability and effectiveness of AI in different academic research types are still insufficient, and issues regarding the ownership of intellectual property, originality, and academic misconduct prevention will be the direction of future research efforts.

### 2. Authorship controversy

ChatGPT’s authorship has been a hot topic since its inception. Several publications have already listed ChatGPT as an author^(45)^. However, many papers have been retracted due to authorship disputes, reflecting widespread misunderstandings, misuse, or abuse of authorship standards at various levels. The academic community is working on comprehensive guidelines aimed at thoroughly assessing the impact of ChatGPT and others on scholarly writing^(56)^ and further clarifying the criteria for defining authorship.

#### 2.1. Consensus and disagreements in the academic world

Some studies indicated that ChatGPT’s technical skills are comparable to human authors^(35^,^57)^, and its rights, privileges, and responsibilities should be recognized. Some publications have already listed ChatGPT as an author^(45)^. The National Institutes of Health (NIH) and the American Heart Association policy mandate the disclosure of ChatGPT use in submissions (professional.heart.org.2024), taking full responsibility for the integrity and authenticity of all generated content^(55)^. The National Science Foundation encourages submitters to state whether and how generative AI were used in developing proposals^(58)^. The ACL 2023 Artificial Intelligence Writing Assistance Policy (https://2023.aclweb.org/blog/ACL-2023-Policy/) provides specific guidance on using generative AI models. The authors should clearly disclose the use of AI-assisted technology before submissions^(59)^ and establish limits to address potential risks, ensuring accuracy and scientific integrity, rather than outright condemning or prohibiting their use in scholarly articles.

Although most scholars acknowledge ChatGPT’s capabilities in writing, they doubt its validity, especially in medical literature^(60^,^61)^. ChatGPT can just serve as an assistant for paper writing and editing or style and language improvement^(44)^.

ChatGPT is found copying others’ intellectual output^(62)^, fabricating references in alarming numbers, lacking accountability and integrity of scientific papers^(63)^, lacking human rationality and responsibility associated with authorship^(64)^, and potentially endangering scientific diversity. It cannot meet the fourth recommendation of the International Committee of Medical Journal Editors regarding authorship and assume the ethical and legal responsibilities associated with its contributions^(43)^.

Some scholars also addressed issues such as contribution level^(63)^, transparency in the publication process^(60)^, data or other content’s compliance with academic integrity^(43)^, whether it can meet the authorship responsibility expectations, and take responsibility for the claims made^(65)^, clearly stating that ChatGPT cannot be listed as an author.

Scholars base their views on ChatGPT’s increasing prevalence and competence or focus on plagiarism, lack of novel perspectives, human rationality, and responsibility associated with authorship. These factors conflict with authorship and may endanger academic writing, scientific diversity, and the integrity of academic publishing. Specific roles, responsibilities, and obligations ChatGPT should assume to ensure the data integrity and authenticity of its generated content should be set.

#### 2.2. Academic ethics and standards for authorship

There has been extensive discussion and various policies regarding recognizing authorship in academic publishing. *JAMA*^(57)^, *Nature, Springer Nature^(60)^, SAGE^(66)^, Frontiers*^(67)^, *Taylor & Francis*^(68)^, and *Wiley*^(69)^ have all placed restrictions, considering ChatGPT inconsistent with journal standards for authors^(6)^. *Science* has banned its use^(43^,^70)^, and *The Lancet Digital Health* has adopted a similar stance. The World Association of Medical Editors, considering the JCMJE standards, also negates its valid authorship^(71)^. The policy of the *Proceedings of the National Academy of Sciences* (PNAS) holds the same stance (https://www.pnas.org/post/update/PNAS-policy-for-ChatGPT-generate-AI); otherwise, it constitutes scientific misconduct (https://www.science.org/content/page/science-journals-editorial-policies).

*Nature* has expressed concerns about scientific transparency, even labeling it a threat^(60)^. ChatGPT has lost its authorship status in at least one paper. The publishing industry calls for clearer policies and guidelines to regulate AI, strengthen fact-checking processes^(72)^, and adopt advanced AI detectors to identify fraudulent use.

Internationally, academic conferences such as the International Machine Learning Conference banned papers containing content generated by ChatGPT. Educational institutions such as the University of Massachusetts Amherst have explicitly prohibited students from using ChatGPT without teacher supervision. The Oxford University Press has also issued guidance statements. Regions such as New York have implemented blocking measures against ChatGPT. The NIH expressly prohibits the use in the peer review process, claiming it violates confidentiality regulations and arouses expert and contextual biases^(73)^.

Several aspects, including effectively integrating AI tools to enhance the efficiency and quality of academic research while ensuring ethics and transparency, remain unaffected. More empirical studies are needed to assess the positive and negative effects of AI tools in academic writing and formulate more comprehensive and specific usage guidelines.

### 3. Ethics and challenges in intelligent writing

ChatGPT applications in writing also trigger a series of ethical issues and challenges. These challenges include content authenticity, data accuracy, bias, and academic integrity violations.

#### 3.1. Data errors and deviation

Scholars pointed out that ChatGPT shows significant deficiencies in handling tasks requiring critical thinking and professional depth, lacking personalized and in-depth insights into specific fields^(47)^. The accuracy and reliability of manuscripts are questioned, with research indicating that these texts may contain misleading and inaccurate information^(62)^. The content output by ChatGPT is often influenced by the quality of its training data^(74)^, which may lead to information bias^(50)^, spurious data^(75)^, and data privacy infringement, affecting its overall reliability. The OpenAI website acknowledges that ChatGPT responses may not be evidence-based.

If users do not provide sufficiently specific requests, AI will assume their requirements, citing fictitious references, raising concerns about credibility^(57)^, and posing challenges for detection. Copyright infringement still occurs. Therefore, data verification and correction by professionals become particularly crucial.

ChatGPT performs inadequately when asked to answer profound questions, often making errors in reasoning and facts, demonstrating limitations^(76)^, or giving meaningless answers, incorrect information, lacking originality and innovation, further limiting its application in academic research. ChatGPT may lack the professional knowledge to assess the scientific validity or accuracy of complex research outcomes, facing difficulties reviewing highly specialized scientific topics^(51)^, especially in generating specific local experimental details and analyzing professional data^(77)^.

#### 3.2. Bias and plagiarism

ChatGPT is not objective or neutral but a powerful tool legitimized by Western automation and efficiency logic^(78)^, potentially producing discriminatory outputs. Data used by ChatGPT may lead to overt or covert biased outputs, reinforce biases^(18)^, or perpetuate existing biases and inequalities.

Numerous studies have shown that inherent gender, racial, and other biases cause AI to reproduce social inequalities and injustices^(79)^. Researchers from the University of California Berkeley Computer Lab discovered biases against nonmale and non-White scientists^(80)^, with outputs potentially using racist and sexist language.

Linguistic bias is also a key challenge^(81)^. ChatGPT is trained to follow English instructions, inappropriately embedding Western values^(60)^ possibly providing unfair or discriminatory arguments or advice to queries from different cultures and/or languages. Nonnative English speakers face a higher frequency of manuscript revision compared to native English speakers^(82)^. The prevalent use of socially biased language in recommendation letters written with the help of ChatGPT is a longstanding issue, particularly in academic medicine and medical education^(83)^, warranting cautious treatment.

Answers and texts generated by ChatGPT are becoming increasingly indistinguishable from those produced by humans^(84)^. Even if researchers do not intend to plagiarize, the likelihood of ChatGPT generating language that is too similar to already published works could lead to unintentional plagiarism^(18)^.

ChatGPT also creates false but realistic datasets, which plagiarism detection software may fail to identify^(75)^, raising concerns about the consistency and accuracy of AI plagiarism checks^(17)^ and worries about ChatGPT research integrity^(8^,^35^,^43)^. Eight universities, including Oxford and Cambridge, have confirmed that using ChatGPT to complete assignments constitutes academic misconduct^(85)^. The Future of Life Institute has also called for a pause in Gen-AI development to buy time for envisioning and safeguarding an AI-driven future.

#### 3.3. Academic integrity challenges

Some scholars have expressed concerns about ChatGPT’s potential to undermine academic integrity. Inaccuracy, irrelevance, specific knowledge deficiency, and lack of original insights remain. Scholars also expressed concerns about academic interiority principles by educational institutions^(19^,^20)^.

Excessive reliance on ChatGPT could potentially lead to the homogenization of writing styles and thought processes^(14)^, resulting in a decline in critical thinking and creative writing abilities^(86)^, causing learning losses and violating academic integrity principles^(14^,^15)^. Misinformation and biases could mislead students, harming knowledge practices and scientific progress^(87)^. ChatGPT’s recommendations might result in inaccurate and varied writing styles^(22)^, making it challenging for teachers to distinguish, threatening educational equity and academic integrity^(12^,^79)^.

ChatGPT fabricates research data or results to meet funding or publication requirements^(88)^. Some scholars believe it will exacerbate financial sustainability, peer reviewer shortages, and global equity in the publishing industry^(89)^. To address these issues, experts actively seek ways to benefit without compromising the ethics of peer review^(48)^. Journals using AI in academic papers should implement strict guidelines and rigorously assess the validity of AI-generated content to limit its misuse.

Cautious and creative adoption of ChatGPT in higher education is crucial. It is essential to involve students, teachers, policymakers, technology developers, and other stakeholders in reviewing and developing strategies^(90)^. Responsible and ethical solutions must be sought to address identified issues and risks. Teachers can update writing tasks and assessment criteria, emphasizing critical thinking and creativity. The effective, ethical, and responsible use of ChatGPT in writing can be promoted through collective efforts.

## DISCUSSION

As one of the most advanced language generation models, ChatGPT has demonstrated immense potential and value in writing. This study gained a comprehensive understanding of the ChatGPT application status in the writing domain by systematically reviewing and analyzing relevant studies in the Web of Science database. Despite some doubts and challenges, the future develop-ment direction remains full of hope and opportunity.

**Enhanced intelligence.** Future research should focus on further optimizing algorithms and models using data from various sources and underrepresented groups, conducting regular audits^(91)^, adjusting and retraining models when biases are detected^(89)^, continuous monitoring and iterative improvements, filtering out false or biased mechanism, and establishing stronger assessment and regulatory mechanisms to ensure the reliability of sources, information quality, and unbiased performance of ChatGPT.

Improving semantic understanding, creativity, and logic will enable ChatGPT to capture the author’s intentions more accurately and generate appropriate and logical content according to the user’s writing habits, style preferences, and demand differences. Promoting algorithm optimization and data accumulation will further enhance generation efficiency and quality to meet writing needs in different scenarios. Emphasizing interaction with users, strengthening human-machine interaction and real-time feedback, adjusting output content, and including editing, proofreading, summarizing, and other functions are crucial.

**Strengthening ethical and legal frameworks.** In the face of issues such as authorship verification, data errors, biases, and privacy protection, future research and development need to strengthen the construction of ethical and legal frameworks to ensure that while improving writing efficiency, data accuracy and academic integrity are guaranteed. More attention should be paid to the transparency and explainability of models, enabling users to understand the AI decision-making process and ensuring the ethical and responsible use of chatbots^(92)^ by establishing stricter ethical guidelines and standards, developing effective identity verification mechanisms, and emphasizing the importance of responsible research that follows ethical, transparent, and evidence-based standards.

**Academic responsibility clarification.** The academic community needs to establish sound frameworks and guidelines^(26)^ to ensure that AI does not obscure the essence of human creativity^(51)^. Researchers must verify and correctly cite information sources to avoid plagiarism^(89)^. Establishing ethical writing practices, strict review processes, and raising awareness of the responsible use of AI are crucial for minimizing the risk of plagiarism.

Clear author responsibility, academic publishing, and peer review policies and standards ensuring fairness, transparency, and accountability are necessary to maintain the safety of the entire publishing ecosystem^(55)^. AI is expected to change peer review by altering participant functions and interactions^(77^,^93)^. By strengthening data protection, content review, and correction mechanisms, intellectual property rights are ensured and comply with academic integrity, ethics, and legal requirements.

The responsibility for detecting text written by AI lies solely with editors and journals/publishers^(94)^, who, as scientific authors and researchers, should rigorously examine research findings and enhance their accuracy in identifying and exposing predatory journals^(95)^. To improve peer review transparency, blockchain technology may be introduced to make the entire review process publicly traceable, combining quantitative metrics with qualitative evaluations to form a more comprehensive academic evaluation system.

**Multilingual interaction and collaboration.** ChatGPT and its derivative technologies can support the generation of multilingual texts. It is necessary to overcome language limitations and biases, promote global knowledge sharing, and drive technological progress and cultural development. Intelligently adjusting language style and habits to suit users from different cultural backgrounds can enhance the efficiency and quality of cross-cultural communication. By building an open and inclusive platform that encourages the sharing of technical challenges and optimizes user experiences, a positive interaction can be fostered, promoting sustainable ChatGPT development in the field of writing.

Although ChatGPT applications still face many challenges in writing, their immense potential cannot be overlooked. Through continuous research and technological innovation, there is reason to believe that ChatGPT and its future iterations will improve writing efficiency, promote knowledge dissemination, and stimulate creative thinking.

## References

[1] Dowling M, Lucey B. (2023). ChatGPT for (finance) research: the Bananarama
conjecture. Fin Res Lett.

[2] Eke DO. (2023). ChatGPT and the rise of generative AI: threat to academic
integrity?. J Responsib Technol.

[3] Lim WM, Gunasekara A, Pallant JL, Pallant JI, Pechenkina E. (2023). Generative AI and the future of education: Ragnarök or
reformation? A paradoxical perspective from management
educators. Int J Manag Educ.

[4] Javaid M, Haleem A, Singh RP. (2023). ChatGPT for healthcare services: an emerging stage for an
innovative perspective. BenchCouncil Transactions on
Benchmarks. Standards and Evaluations.

[5] Taecharungroj V. (2023). What can ChatGPT do? Analyzing early reactions to the innovative
AI chatbot on Twitter. Big Data Cogn Comput.

[6] Salvagno M, Taccone FS, Gerli AG. (2023). Can artificial intelligence help for scientific
writing?. Crit Care.

[7] Nordling L. (2023). How ChatGPT is transforming the postdoc
experience. Nature.

[8] Else H. (2023). Abstracts written by ChatGPT fool scientists. Nature.

[9] Biswas S. (2023). ChatGPT and the future of medical writing. Radiology.

[10] Hubert KF, Awa KN, Zabelina DL. (2024). The current state of artificial intelligence generative language
models is more creative than humans on divergent thinking
tasks. Sci Rep.

[11] Meunier F, Pikhart M, Klimova B. (2022). Editorial: new perspectives of L2 acquisition related to
human-computer interaction (HCI). Front Psychol.

[12] Yan D. (2023). Impact of ChatGPT on learners in a L2 writing practicum: an
exploratory investigation. Educ Inf Technol.

[13] Zhang R, Zou D, Cheng G. (2023). Chatbot-based learning of logical fallacies in EFL writing:
perceived effectiveness in improving target knowledge and learner
motivation. Interact Learn Environ.

[14] Barrot JS. (2023). Using ChatGPT for second language writing: pitfalls and
potentials. Assess Writ.

[15] Su Y, Lin Y, Lai C. (2023). Collaborating with ChatGPT in argumentative writing
classrooms. Assess Writ.

[16] Ansari AN, Ahmad S, Bhutta SM. (2024). Mapping the global evidence around the use of ChatGPT in higher
education: a systematic scoping review. Educ Inf Technol.

[17] Cotton DR, Cotton PA, Shipway JR. (2024). Chatting and cheating: ensuring academic integrity in the era of
ChatGPT. Innov Educ Teach Int.

[18] Dalalah D, Dalalah OM. (2023). The false positives and false negatives of generative AI
detection tools in education and academic research: the case of
ChatGPT. Int J Manag Educ.

[19] Guo K, Wang J, Chu SK. (2022). Using chatbots to scaffold EFL students’ argumentative
writing. Assess Writ.

[20] Kasneci E, Sessler K, Küchemann S, Bannert M, Dementieva D, Fischer F (2023). ChatGPT for good? On opportunities and challenges of large
language models for education. Learn Individ Differ.

[21] Zou M, Huang L. (2023). To use or not to use? Understanding doctoral students’ acceptance
of ChatGPT in writing through technology acceptance model. Front Psychol.

[22] Song C, Song Y. (2023). Enhancing academic writing skills and motivation: assessing the
efficacy of ChatGPT in AI-assisted language learning for EFL
students. Front Psychol.

[23] Nazari N, Shabbir MS, Setiawan R. (2021). Application of Artificial Intelligence powered digital writing
assistant in higher education: randomized controlled trial. Heliyon.

[24] Hwang WY, Nurtantyana R, Purba SW, Hariyanti U, Indrihapsari Y, Surjono HD. (2023). AI and recognition technologies to facilitate English as foreign
language writing for supporting personalization and contextualization in
authentic contexts. J Educ Comput Res.

[25] Ivanov S, Soliman M. (2023). Game of algorithms: ChatGPT implications for the future of
tourism education and research. J Tourism Futures.

[26] Huang J, Tan M. (2023). The role of ChatGPT in scientific communication: writing better
scientific review articles. Am J Cancer Res.

[27] Alkaissi H, McFarlane SI. (2023). Artificial hallucinations in ChatGPT: implications in scientific
writing. Cureus.

[28] Humphry T, Fuller AL. (2023). Potential ChatGPT use in undergraduate chemistry
laboratories. J Chem Educ.

[29] Koo M. (2023). The importance of proper use of ChatGPT in medical
writing. Radiology.

[30] Noy S, Zhang W. (2023). Experimental evidence on the productivity effects of generative
artificial intelligence. Science.

[31] Abdullayeva M, Musayeva ZM. (2023). The impact of ChatGPT on students’ writing skills: an exploration
of AI-assisted writing tools. International Conference of Education, Research and Innovation.

[32] Rahimi F, Talebi Bezmin Abadi A (2023). Passive contribution of ChatGPT to scientific
papers. Ann Biomed Eng.

[33] Arouse A. (2023). Overcoming the language barrier in science
communication. Nat Rev Bioeng.

[34] González-Arias C, López-García X. (2023). ChatGPT: stream of opinion in five newspapers in the first 100
days since its launch. Prof Inf.

[35] Sallam M. (2023). ChatGPT utility in healthcare education, research, and practice:
systematic review on the promising perspectives and valid
concerns. Healthcare (Basel).

[36] Stokel-Walker C. (2022). AI bot ChatGPT writes smart essays - should professors
worry?. Nature.

[37] Carobene A, Padoan A, Cabitza F, Banfi G, Plebani M. (2024). Rising adoption of artificial intelligence in scientific
publishing: evaluating the role, risks, and ethical implications in paper
drafting and review process [CCLM]. Clin Chem Lab Med.

[38] Šedlbauer J, Činčera J, Slavík M, Hartlová A. (2024). Students’ reflections on their experience with
ChatGPT. J Comput Assist Learn.

[39] Rice S, Crouse SR, Winter SR, Rice C. (2024). The advantages and limitations of using ChatGPT to enhance
technological research. Technol Soc.

[40] Chubb LA. (2023). Me and the machines: possibilities and pitfalls of using
artificial intelligence for qualitative data analysis. Int J Qual Methods.

[41] Babl FE, Babl MP. (2023). Generative artificial intelligence: can ChatGPT write a quality
abstract?. Emerg Med Australas.

[42] Berdejo-Espinola V, Amano T. (2023). AI tools can improve equity in science. Science.

[43] Thorp HH. (2023). ChatGPT is fun, but not an author. Science.

[44] Kayaalp ME, Ollivier M, Winkler PW, Dahmen J, Musahl V, Hirschmann MT (2024). Embrace responsible ChatGPT usage to overcome language barriers
in academic writing. Knee Surg Sports Traumatol Arthrosc.

[45] Tufano R, Dabić O, Mastropaolo A, Ciniselli M, Bavota G. (2024). Code review automation: strengths and weaknesses of the state of
the art. IEEE Trans Softw Eng.

[46] Curtis N. (2023). To ChatGPT or not to ChatGPT? The impact of artificial
intelligence on academic publishing. Pediatr Infect Dis J.

[47] Chen T, Wang X, Yue T, Bai X, Le CX, Wang W. (2023). Enhancing abstractive summarization with extracted knowledge
graphs and multi-source transformers. Appl Sci (Basel).

[48] Hosseini M, Rasmussen LM, Resnik DB. (2024). Using AI to write scholarly publications. Account Res.

[49] Hosseini M, Horbach SP. (2023). Fighting reviewer fatigue or amplifying bias? Considerations and
recommendations for use of ChatGPT and other large language models in
scholarly peer review. Res Integr Peer Rev.

[50] Biswas S, Dobaria D, Cohen HL. (2023). ChatGPT and the future of journal reviews: A feasibility
study. Yale J Biol Med.

[51] Koga S. (2023). The integration of large language models such as ChatGPT in
scientific writing: harnessing potential and addressing
pitfalls. Korean J Radiol.

[52] Pal S, Bhattacharya M, Islam MA, Chakraborty C. (2024). AI-enabled ChatGPT or LLM: a new algorithm is required for
plagiarism-free scientific writing. Int J Surg.

[53] Brainard J. (2023). New tools show promise for tackling paper mills. Science.

[54] Hung J, Chen J. (2023). The benefits, risks and regulation of using ChatGPT in Chinese
academia: a content analysis. Soc Sci (Basel).

[55] Inam M. (2024). AHA Postdoctoral Fellowship. Professional Heart Daily.

[56] Cacciamani GE, Collins GS, Gill IS. (2023). ChatGPT: standard reporting guidelines for responsible
use. Nature.

[57] Flanagin A, Bibbins-Domingo K, Berkwits M, Christiansen SL. (2023). Nonhuman “authors” and implications for the integrity of
scientific publication and medical knowledge. JAMA.

[58] Notice to Research Community (2023). Use of generative artificial intelligence technology in the NSF
merit review process. NSF-National Science Foundation.

[59] International Committee of Medical Journals Editors (ICMJE) (2023). Defining the role of authors and contributors.

[60] (2023). Tools such as ChatGPT threaten transparent science; here are our
ground rules for their use [editorial]. Nature.

[61] Stokel-Walker C, Van Noorden R. (2023). What ChatGPT and generative AI mean for science. Nature.

[62] Lund BD, Wang T, Mannuru NR, Nie B, Shimray S, Wang Z. (2023). ChatGPT and a new academic reality: artificial
Intelligence-written research papers and the ethics of the large language
models in scholarly publishing. J Assoc Inf Sci Technol.

[63] Stokel-Walker C. (2023). ChatGPT listed as author on research papers: many scientists
disapprove. Nature.

[64] Silva JAT, Tsigaris P. (2023). Humanand AI -based authorship: principles and
ethics. Learn Publ.

[65] Kim SG. (2023). Using ChatGPT for language editing in scientific
articles. Maxillofac Plast Reconstr Surg.

[66] Artificial Intelligence Policy (2023). SAGE.

[67] Author Guidelines-Writing and formating https://www.frontiersin.org/guidelines/author-guidelines.

[68] Taylor & Francis Clarifies the responsible use of AI tools in
academic content creation (2023). https://newsroom.taylorandfrancisgroup.com/taylor-francis-clarifies-the-responsible-use-of-ai-tools-in-academic-content-creation/.

[69] Artificial intelligence generated content (2023). https://authorservices.wiley.com/ethics-guidelines/index.html.

[70] Science Journal’s Editorial Policies (2023). Science;.

[71] Fulton JS. (2023). Authorship and ChatGPT. Clin Nurse Spec.

[72] Seckel E, Stephens BY, Rodriguez F. (2024). Ten simple rules to leverage large language models for getting
grants. PLOS Comput Biol.

[73] Lauer M, Constant S, Wernimont A. (2023). Using AI in peer review is a breach of
confidentiality. NIH Extramural Nexus.

[74] Lee H. (2024). The rise of ChatGPT: exploring its potential in medical
education. Anat Sci Educ.

[75] Naddaf M. (2023). ChatGPT generates fake data set to support scientific
hypothesis. Nature.

[76] Duong D, Solomon BD. (2024). Analysis of large-language model versus human performance for
genetics questions. Eur J Hum Genet.

[77] West JK, Franz JL, Hein SM, Leverentz-Culp HR, Mauser JF, Ruff EF (2023). An analysis of AI-generated laboratory reports across the
chemistry curriculum and student perceptions of ChatGPT. J Chem Educ.

[78] Markowitz DM. (2024). Can generative AI infer thinking style from language? Evaluating
the utility of AI as a psychological text analysis tool. Behav Res Methods.

[79] Dwivedi YK, Kshetri N, Hughes L, Slade EL, Jeyaraj A, Kar AK (2023). So what if ChatGPT wrote it? Multidisciplinary perspectives on
opportunities, challenges and implications of generative conversational AI
for research, practice and policy. Int J Inf Manag.

[80] Alba D. (2022). OpenAI chatbot spits out biased musings, despite
guardrails. Bloomberg.

[81] Seghier ML. (2023). ChatGPT: not all languages are equal. Nature.

[82] Amano T, Ramírez-Castañeda V, Berdejo-Espinola V, Borokini I, Chowdhury S, Golivets M (2023). The manifold costs of being a non-native English speaker in
science. PLoS Biol.

[83] Khan S, Kirubarajan A, Shamsheri T, Clayton A, Mehta G. (2023). Gender bias in reference letters for residency and academic
medicine: a systematic review. Postgrad Med J.

[84] Eaton SE. (2023). Postplagiarism: transdisciplinary ethics and integrity in the age
of artificial intelligence and neurotechnology. Int J Educ Integr.

[85] Chan CK. (2023). A comprehensive AI policy education framework for university
teaching and learning. Int J Educ Technol High Educ.

[86] Zhai X, Nyaaba M, Ma W. (2024). Can generative AI and ChatGPT outperform humans on
cognitive-demanding problem-solving tasks in science?. Sci Educ.

[87] van Dis EA, Bollen J, Zuidema W, van Rooij R, Bockting CL. (2023). ChatGPT: five priorities for research. Nature.

[88] Cascella M, Montomoli J, Bellini V, Bignami E. (2023). Evaluating the feasibility of ChatGPT in healthcare: an analysis
of multiple clinical and research scenarios. J Med Syst.

[89] Page AJ, Tumelty NM, Sheppard SK. (2023). Navigating the AI frontier: ethical considerations and best
practices in microbial genomics research. Microb Genom.

[90] ChatGPT and artificial intelligence in higher education (2023). https://unesdoc.unesco.org/ark:/48223/pf0000385146.

[91] Hassani H, Silva ES. (2023). The role of ChatGPT in data science: how ai-assisted
conversational interfaces are revolutionizing the field. Big Data Cogn Comput.

[92] McGee RW. (2023). Is ChatGPT biased against conservatives? An empirical
study. SSRN Electron J.

[93] Conroy G. (2023). How ChatGPT and other AI tools could disrupt scientific
publishing. Nature.

[94] Teixeira da Silva JA (2023). ChatGPT: detection in academic journals is editors’ and
publishers’ responsibilities. Ann Biomed Eng.

[95] Al-Moghrabi D, Abu Arqub S, Maroulakos MP, Pandis N, Fleming PS. (2024). Can ChatGPT identify predatory biomedical and dental journals? A
cross-sectional content analysis. J Dent.

